# Association of systemic immune-inflammation index (SII) with 28-day all-cause mortality in acute heart failure: A retrospective cohort study

**DOI:** 10.1097/MD.0000000000048027

**Published:** 2026-03-13

**Authors:** Chengyu Zhang, Ziqi Zhang, Zi Yan, HangKai Cui, Xiaonan Ji, Jiangan Li

**Affiliations:** a Wuxi School of Medicine, Jiangnan University, Wuxi, China; b State Key Laboratory of Traditional Chinese Medicine Syndrome, Guangzhou University of Chinese Medicine, Guangzhou, China; c Department of Urology, Sun Yat-Sen Memorial Hospital, Sun Yat-Sen University, Guangzhou, China; d School of Clinical Medicine, Shandong Second Medical University, Weifang, China; e Department of Emergency, Jiangnan University Medical Center, Wuxi, PR China; f Department of Neurosurgery, Jiangnan University Medical Center, Wuxi, PR China; g Department of Emergency, Wuxi No. 2 People’s Hospital, Affiliated Wuxi Clinical College of Nantong University, Wuxi, China.

**Keywords:** acute heart failure (AHF), MIMIC-IV database, prognostic biomarker, retrospective cohort study, systemic immune-inflammation index (SII)

## Abstract

This study aimed to investigate the association between the systemic immune-inflammation index (SII) and 28-day all-cause mortality in patients with acute heart failure (AHF) and to evaluate the potential of SII as a prognostic biomarker for early risk stratification. A retrospective cohort study was conducted using the Medical Information Mart for Intensive Care IV database. Patients with an AHF diagnosis on first intensive care unit admission were included, while those with intensive care unit stays <24 hours or missing key laboratory data were excluded. The SII was calculated using the formula: (platelet count × neutrophil count)/lymphocyte count from initial admission laboratory values. Patients were categorized into tertiles based on their SII values. Kaplan–Meier survival analysis and multivariable Cox proportional hazards models were used to assess the association between SII and 28-day mortality, adjusting for demographic, clinical, and laboratory covariates. A total of 5482 patients were included. Kaplan–Meier analysis showed significant differences in 28-day mortality across SII tertiles (log-rank *P* < .001). In multivariable Cox models, compared with the lowest tertile, the middle and highest SII tertiles were associated with higher 28-day mortality (hazard ratio = 1.485, 95% confidence interval: 1.193–1.849 and hazard ratio = 2.497, 95% confidence interval: 2.060–3.028; both *P* < .01). Similar associations were observed for 90-, 180-, and 365-day mortality. Restricted cubic spline analyses suggested a dose–response relationship between SII and 28-day mortality. In addition, adding SII to Acute Physiology Score III and Sequential Organ Failure Assessment improved discrimination for 28-day mortality in receiver operating characteristic analyses. Elevated SII is an independent predictor of increased 28-day all-cause mortality in AHF patients. As a readily available and cost-effective marker, SII could be integrated into early risk stratification protocols to guide personalized therapeutic strategies in acute care settings.

**Key points**

- Systemic immune-inflammation index (SII) predicts 28-day mortality in AHF.
- Higher SII levels are linked to poor short- and long-term survival outcomes.
- SII improves risk prediction when combined with SOFA and APS III scores.
- SII offers a cost-effective, rapidly available biomarker for emergency triage.
- Elevated SII reflects immune, inflammatory, and thrombotic imbalance in AHF.

## 1. Introduction

Acute heart failure (AHF) represents a critical clinical syndrome frequently encountered in emergency departments, pathologically characterized by acute impairment of ventricular filling or ejection capacity, resulting in dyspnea and markedly diminished exercise tolerance.^[1]^ As a major global public health challenge, heart failure (HF) exhibits persistently elevated incidence, mortality, and rehospitalization rates, with epidemiological estimates indicating a disease burden exceeding 64 million individuals worldwide.^[2]^ Notably, HF demonstrates a 5-year mortality rate surpassing 50%^[3]^ and frequently manifests as the terminal stage of diverse cardiovascular pathologies,^[3]^ thereby underscoring the imperative to establish precise prognostic frameworks for optimizing clinical management. In this context, the identification and validation of prognostically significant biomarkers have emerged as a research priority, offering advantages through cost-effective, minimally invasive, and objective quantification of disease severity to facilitate early risk stratification and personalized therapeutic interventions.^[4]^

While the precise pathogenesis of HF remains incompletely elucidated, contemporary research has established the pivotal regulatory role of immune activation and systemic inflammatory cascades in disease progression.^[5]^ Postinjury myocardial repair and remodeling processes involve intricate networks of immunomodulation, encompassing neutrophils, lymphocytes, and inflammatory mediators such as tumor necrosis factor-alpha and interleukin-6. Of particular clinical relevance, patients with systemic inflammatory disorders demonstrate significantly elevated cardiovascular risk profiles compared with the general population,^[6]^ suggesting the potential utility of inflammatory biomarkers as novel predictors of cardiovascular outcomes.

The systemic immune-inflammation index (SII), derived from integrated platelet, lymphocyte, and neutrophil counts, provides a composite measure of thrombocytosis, inflammatory status, and immune homeostasis. Accumulating evidence demonstrates significant associations between elevated SII levels and cardiovascular morbidity/mortality. Recent studies have reported that SII predicts adverse ventricular remodeling after myocardial infarction, new-onset HF following ST-segment elevation myocardial infarction, and major adverse cardiovascular events in patients with coronary artery disease and HF.^[6–12]^ Although the mechanistic pathways linking SII to HF prognosis remain incompletely defined, this biomarker is postulated to reflect the dynamic interplay between inflammatory activity and immune stress responses under pathological conditions.^[13]^

Current investigations into SII-HF prognostic correlations remain limited by population heterogeneity and methodological constraints. Although SII has been linked to cardiovascular prognosis in chronic HF and acute coronary syndromes, evidence regarding its role in short-term outcomes in AHF remains limited, particularly in critically ill populations.^[14]^ To address this knowledge gap, we conducted a comprehensive analysis utilizing the Medical Information Mart for Intensive Care IV (MIMIC-IV) database to elucidate the association between SII levels and 28-day all-cause mortality in AHF patients, with the ultimate objective of advancing evidence-based strategies for early risk stratification and precision medicine in AHF management.^[15]^

## 2. Materials and methods

### 2.1. Data source and study population

**Figure 1.**
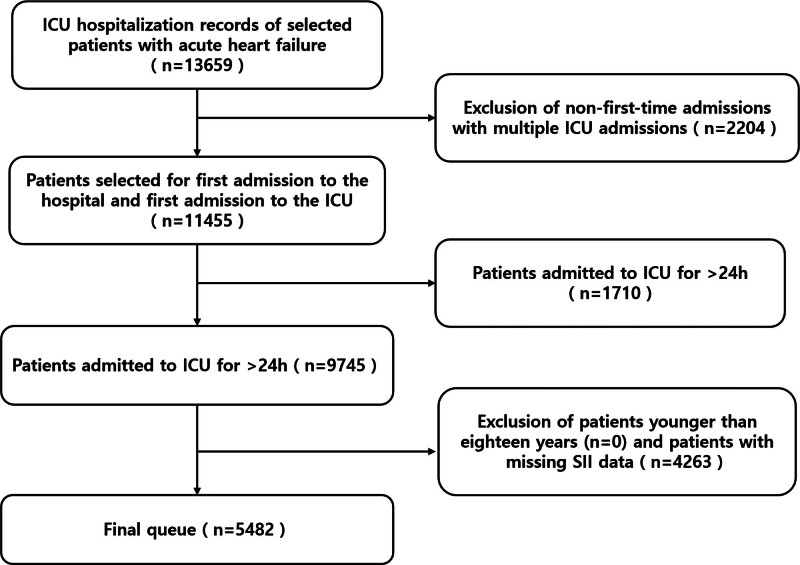
Flowchart showing the selection process of intensive care unit (ICU) patients with acute heart failure for the study. ICU = intensive care unit, SII = systemic immune-inflammation index.

We performed a retrospective cohort analysis utilizing clinical data extracted from the MIMIC-IV database, a publicly available repository containing deidentified health records of over 50,000 intensive care unit (ICU) admissions at Beth Israel Deaconess Medical Center between 2008 and 2019. Authorization for data access was secured by the first author Chengyu Zhang (Record ID: 13849257). From an initial pool of 11,455 patients, we applied 2 inclusion criteria: AHF diagnosis per implantable cardioverter-defibrillator (ICD)-9/ICD-10 codes –AHF was identified using ICD-9 and ICD-10 diagnostic codes recorded at ICU admission. This approach has been commonly used in MIMIC-based observational studies. The second inclusion criteria was first-time hospitalization with initial ICU admission. Exclusion criteria comprised ICU stays <24 hours (n = 1710) and missing admission platelet/neutrophil/lymphocyte counts (n = 4263). The final analytical cohort included 5482 patients stratified by SII tertiles, and the participant selection process is illustrated in Figure 1. Due to the lack of systematically available echocardiographic data in the database, further classification of AHF into reduced or preserved ejection fraction subtypes was not performed.

### 2.2. Study variables

The SII was calculated using the formula: SII = (platelet count × neutrophil count)/lymphocyte count, based on initial postadmission laboratory measurements. The primary endpoint was defined as 28-day all-cause mortality, with secondary endpoints including 90-, 180-, and 365-day mortality outcomes.

### 2.3. Covariates

Covariate extraction from MIMIC-IV employed PostgreSQL (v17.2; PostgreSQL Global Development Group, Berkeley), encompassing demographic variables (age, sex, and ethnicity), laboratory parameters (white blood cell [WBC], red blood cell, platelets, hemoglobin, red cell distribution width [RDW], hematocrit, electrolytes, coagulation markers, and renal function tests), disease severity scores (Sequential Organ Failure Assessment [SOFA], Acute Physiology Score III [APS III], Oxford Acute Severity of Illness Score [OASIS], and systemic inflammatory response syndrome), comorbidities (hypertension, diabetes, chronic obstructive pulmonary disease [COPD], myocardial infarction, atrial fibrillation, cerebrovascular disease, dyslipidemia, hepatic dysfunction, malignancy, coronary artery disease, chronic kidney disease, and cardiomyopathy), pharmacological treatments (angiotensin-converting enzyme inhibitors/angiotensin II receptor blockers, diuretics, statins, and antiplatelet/anticoagulant agents), and procedural interventions (coronary artery bypass grafting, percutaneous coronary intervention, and ICD implantation). Continuous variables exceeding 20% missing data were excluded, while limited missing values (<20%) underwent *k*-nearest neighbors imputation via the DMwR2 package in R (R Foundation for Statistical Computing, Vienna, Austria).

### 2.4. Statistical analyses

Categorical variables were presented as frequencies (%) with χ^2^ test comparisons, while continuous variables were expressed as mean ± standard deviation and analyzed through *t* tests/analysis of variance. Survival differences across SII tertiles were assessed via Kaplan–Meier curves with log-rank testing. Cox proportional hazards models evaluated SII-mortality associations after verifying proportional hazards assumptions through Schoenfeld residuals and controlling multicollinearity (variance inflation factor < 5). Four adjustment models were implemented: model 1 (unadjusted), model 2 (age/sex-adjusted), model 3 (additional comorbidity adjustment), and model 4 (medication/intervention adjustment). Restricted cubic splines (RCSs) characterized nonlinear SII-mortality relationships, supplemented by subgroup analyses and sensitivity testing excluding malignancy cases. All analyses were conducted in R v4.1.3 (R Foundation for Statistical Computing, Vienna, Austria), with 2-tailed *P* < .05 denoting statistical significance.

## 3. Results

### 3.1. Baseline characteristics

Table 1 delineates baseline characteristics of 5482 patients stratified by SII tertiles (Q1 ≤ 886.57; 886.57 < Q2 ≤ 2118.25; Q3 > 2118.25). The cohort had a mean age of 71.00 (13.68) years, with males accounting for 56.3% (*P* = .662). Higher SII tertiles correlated with older age (*P* < .001), elevated WBC, platelet, neutrophil, RDW, anion gap, prothrombin time, international normalized ratio, blood urea nitrogen, creatinine, APS III, OASIS scores, and severe systemic inflammatory response syndrome (all *P* < .05), as well as reduced lymphocyte count and chloride (*P* < .001). Upper tertiles showed higher prevalence of COPD, renal disease, malignant cancer, and lower hypertension, angiotensin-converting enzyme inhibitor/angiotensin II receptor blocker use (*P* < .05); diabetes and myocardial infarction had no upper-tertile increment (*P* = .005, *P* = .533). Hospital/ICU length of stay and 28-/90-/180-/365-day mortality were higher in Q3 (*P* < .001*). No significant differences existed for sex, red blood cell, hemoglobin, sodium, potassium, partial thromboplastin time, SOFA score, cerebrovascular disease, or anticoagulant use (*P > .05).

**Table 1:** Baseline characteristics of participants stratified by SII tertiles.

	Total (n = 5482)	SII tertiles			*P*-value
		Q1 (n = 1828)	Q2 (n = 1827)	Q3 (n = 1827)	
Age, yr	71.00 (13.68)	69.87 (14.20)	71.24 (13.50)	71.90 (13.23)	<.001
Gender, n (%)					.662
Male	3089 (56.3)	1015 (55.5)	1041 (57.0)	1033 (56.5)	
Female	2393 (43.7)	813 (44.5)	786 (43.0)	794 (43.5)	
Length of stay (LOS), d	16.06 ± 14.80	15.04 ± 14.74	15.84 ± 13.76	17.30 ± 15.75	<.001
ICU length of stay (ICULOS), d	5.20 ± 6.10	4.69 ± 5.76	5.13 ± 5.86	5.79 ± 6.58	<.001
WBC, K/μL	12.61 ± 8.31	10.75 ± 9.70	11.98 ± 5.92	15.10 ± 8.25	<.001
RBC, m/μL	3.53 ± 0.83	3.48 ± 0.85	3.54 ± 0.82	3.56 ± 0.82	.015
Platelet, K/μL	211.71 ± 97.82	164.11 ± 67.15	209.98 ± 79.77	261.06 ± 114.62	<.001
Neutrophil count, K/μL	9.57 ± 6.50	5.96 ± 3.93	8.92 ± 4.73	13.83 ± 7.56	<.001
Lymphocyte count, K/μL	11.19 ± 176.25	31.46 ± 304.26	1.31 ± 0.76	0.80 ± 0.55	<.001
RDW, %	15.91 ± 2.56	15.65 ± 2.48	15.85 ± 2.55	16.23 ± 2.62	<.001
Hemoglobin, g/dL	10.32 ± 2.61	10.36 ± 2.38	10.33 ± 2.31	10.26 ± 3.07	.471
Sodium, mmol/L	137.41 ± 4.24	137.46 ± 4.04	137.35 ± 4.26	137.41 ± 4.42	.719
Potassium, mmol/L	4.27 ± 0.57	4.25 ± 0.58	4.27 ± 0.56	4.29 ± 0.58	.083
Chloride, mmol/L	100.68 ± 6.46	101.67 ± 6.13	100.61 ± 6.51	99.77 ± 6.58	<.001
Anion gap, mmol/L	14.95 ± 4.14	14.35 ± 3.92	14.80 ± 3.95	15.69 ± 4.40	<.001
PT, s	18.21 ± 10.57	17.77 ± 9.37	17.93 ± 10.00	18.94 ± 12.11	.001
PTT, s	36.38 ± 12.72	36.20 ± 12.28	36.54 ± 13.14	36.39 ± 12.73	.728
INR	1.64 ± 0.78	1.61 ± 0.73	1.61 ± 0.76	1.68 ± 0.85	.006
Urea nitrogen, mg/dL	37.37 ± 26.49	33.12 ± 23.49	36.86 ± 26.38	42.15 ± 28.58	<.001
CRE, mg/dL	1.87 ± 1.62	1.79 ± 1.68	1.82 ± 1.51	2.01 ± 1.64	<.001
SOFA	5.47 ± 3.28	5.63 ± 3.31	5.15 ± 3.16	5.63 ± 3.36	<.001
APSIII	48.31 ± 17.64	46.07 ± 17.62	47.33 ± 16.89	51.53 ± 17.96	<.001
OASIS	32.10 ± 8.24	31.11 ± 8.09	31.65 ± 8.17	33.53 ± 8.28	<.001
SIRS, n (%)					<.001
0	38 (0.7)	19 (1.0)	14 (0.8)	5 (0.3)	
1	659 (12.0)	266 (14.6)	232 (12.7)	161 (8.8)	
2	1977 (36.1)	717 (39.2)	705 (38.6)	555 (30.4)	
3	2147 (39.2)	619 (33.9)	690 (37.8)	838 (45.9)	
4	661 (12.1)	207 (11.3)	186 (10.2)	268 (14.7)	
Hypertension, n (%)	781 (14.2)	325 (17.8)	267 (14.6)	189 (10.3)	<.001
Diabetes, n (%)	2568 (46.8)	869 (47.5)	897 (49.1)	802 (43.9)	.005
Atrial fibrillation, n (%)	3027 (55.2)	949 (51.9)	1033 (56.5)	1045 (57.2)	.002
Dyslipidemia, n (%)	2885 (52.6)	936 (51.2)	1002 (54.8)	947 (51.8)	.062
Myocardial infarction, n (%)	2039 (37.2)	661 (36.2)	689 (37.7)	689 (37.7)	.533
Cerebrovascular disease, n (%)	659 (12.0)	203 (11.1)	242 (13.2)	214 (11.7)	.122
COPD, n (%)	1952 (35.6)	596 (32.6)	635 (34.8)	721 (39.5)	<.001
Liver disease, n (%)	525 (9.6)	209 (11.4)	156 (8.5)	160 (8.8)	.004
Renal disease, n (%)	2544 (46.4)	799 (43.7)	856 (46.9)	889 (48.7)	.01
Malignant cancer, n (%)	520 (9.5)	169 (9.2)	146 (8.0)	205 (11.2)	.004
Coronary artery disease, n (%)	2760 (50.3)	900 (49.2)	955 (52.3)	905 (49.5)	.129
Cardiomyopathy, n (%)	1490 (27.2)	516 (28.2)	528 (28.9)	446 (24.4)	.004
Implantable cardioverter-defibrillator pacemaker, n (%)	313 (5.7)	126 (6.9)	115 (6.3)	72 (3.9)	<.001
Percutaneous coronary intervention, n (%)	484 (8.8)	180 (9.8)	153 (8.4)	151 (8.3)	.17
Coronary bypass operation, n (%)	549 (10.0)	256 (14.0)	201 (11.0)	92 (5.0)	<.001
Used diuretic, n (%)	5041 (92.0)	1671 (91.4)	1704 (93.3)	1666 (91.2)	.04
Used antiplatelet, n (%)	3925 (71.6)	1309 (71.6)	1344 (73.6)	1272 (69.6)	.031
Used anticoagulant, n (%)	5190 (94.7)	1722 (94.2)	1728 (94.6)	1740 (95.2)	.369
Used statin, n (%)	3655 (66.7)	1181 (64.6)	1264 (69.2)	1210 (66.2)	.012
Used ACEI/ARB, n (%)	2448 (44.7)	941 (51.5)	843 (46.1)	664 (36.3)	<.001
180-d readmission, n (%)	1398 (25.5)	500 (27.4)	474 (25.9)	424 (23.2)	.014
Death within 28 d, n (%)	767 (14.0)	141 (7.7)	206 (11.3)	420 (23.0)	<.001
Death within 90 d, n (%)	1250 (22.8)	252 (13.8)	365 (20.0)	633 (34.6)	<.001
Death within 180 d, n (%)	1531 (27.9)	338 (18.5)	468 (25.6)	725 (39.7)	<.001
Death within 365 d, n (%)	1913 (34.9)	455 (24.9)	604 (33.1)	854 (46.7)	<.001

ACEI/ARB = angiotensin-converting enzyme inhibitor/angiotensin II receptor blocker, APS III = Acute Physiology Score III, COPD = chronic obstructive pulmonary disease, CRE = creatinine, ICU = intensive care unit, INR = international normalized ratio, OASIS = Oxford Acute Severity of Illness Score, PT = prothrombin time, PTT = partial thromboplastin time, RBC = red blood cell, RDW = red cell distribution width, SII = systemic immune-inflammation index, SIRS = systemic inflammatory response syndrome, SOFA = Sequential Organ Failure Assessment, WBC = white blood cell.

Table 2 compares 28-day survivors (n = 4715) and nonsurvivors (n = 767). Nonsurvivors had higher SII (implied), age, male proportion, WBC, neutrophil, RDW, anion gap, prothrombin time, partial thromboplastin time, international normalized ratio, urea nitrogen, creatinine, SOFA, APS III, OASIS scores (all *P* < .05), lower platelet, hemoglobin, chloride, and 180-day readmission (*P* < .05); female proportion was lower in nonsurvivors. Survivors had higher hypertension, diuretic/antiplatelet/statin use, and intervention rates (*P* < .05); dyslipidemia showed no difference (*P* = .947). Survivors had lower prevalence of atrial fibrillation, myocardial infarction, cerebrovascular/liver/renal disease, and malignant cancer (*P* < .05); COPD had no difference (*P* = .204).

**Table 2:** Baseline characteristics of participants by 28-day survival status.

	Total (n = 5482)	Survival (n = 4715)	Nonsurvival (n = 767)	*P*-value
Age, yr (mean [SD])	71.00 (13.68)	70.18 (13.75)	76.04 (12.05)	<.001
Gender, n (%)				
Male	3089 (56.3)	2622 (55.6)	467 (60.9)	.007
Female	2393 (43.7)	2093 (44.4)	300 (39.1)	
Length of stay (LOS), d	16.06 (14.80)	16.58 (15.37)	12.85 (10.03)	<.001
ICU length of stay (ICULOS), d	5.20 (6.10)	5.02 (6.23)	6.34 (5.07)	<.001
WBC, K/μL	12.61 (8.31)	12.38 (8.10)	14.04 (9.37)	<.001
RBC, m/μL	3.53 (0.83)	3.55 (0.83)	3.40 (0.80)	<.001
Platelet, K/μL	211.71 (97.82)	213.03 (96.65)	203.60 (104.40)	.013
Neutrophil count, K/μL	9.57 (6.50)	9.13 (6.10)	12.26 (8.04)	<.001
Lymphocyte count, K/μL	11.19 (176.25)	11.89 (183.76)	6.90 (120.15)	.467
RDW, %	15.91 (2.56)	15.76 (2.48)	16.81 (2.86)	<.001
Hemoglobin, g/dL	10.32 (2.61)	10.39 (2.66)	9.89 (2.24)	<.001
Sodium, mmol/L	137.41 (4.24)	137.36 (4.17)	137.70 (4.68)	.039
Potassium, mmol/L	4.27 (0.57)	4.27 (0.57)	4.31 (0.60)	.067
Chloride, mmol/L	100.68 (6.46)	100.85 (6.40)	99.64 (6.73)	<.001
Anion gap, mmol/L	14.95 (4.14)	14.72 (3.99)	16.31 (4.72)	<.001
PT, s	18.21 (10.57)	17.75 (9.82)	21.03 (14.03)	<.001
PTT, s	36.38 (12.72)	36.10 (12.54)	38.06 (13.66)	<.001
INR	1.64 (0.78)	1.60 (0.75)	1.85 (0.95)	<.001
Urea nitrogen, mg/dL	37.37 (26.49)	35.46 (25.27)	49.13 (30.48)	<.001
CRE, mg/dL	1.87 (1.62)	1.82 (1.60)	2.23 (1.66)	<.001
SOFA	5.47 (3.28)	5.19 (3.14)	7.21 (3.61)	<001
APSIII	48.31 (17.64)	46.55 (16.65)	59.14 (19.64)	<.001
OASIS	32.10 (8.24)	31.44 (8.04)	36.14 (8.35)	<.001
SIRS, n (%)				<.001
0	38 (0.7)	35 (0.7)	3 (0.4)	
1	659 (12.0)	588 (12.5)	71 (9.3)	
2	1977 (36.1)	1763 (37.4)	214 (27.9)	
3	2147 (39.2)	1813 (38.5)	334 (43.5)	
4	661 (12.1)	516 (10.9)	145 (18.9)	
Hypertension, n (%)	781 (14.2)	726 (15.4)	55 (7.2)	<.001
Diabetes, n (%)	2568 (46.8)	2217 (47.0)	351 (45.8)	.543
Atrial fibrillation, n (%)	3027 (55.2)	2533 (53.7)	494 (64.4)	<.001
Dyslipidemia, n (%)	2885 (52.6)	2480 (52.6)	405 (52.8)	.947
Myocardial infarction, n (%)	2039 (37.2)	1690 (35.8)	349 (45.5)	<.001
Cerebrovascular disease, n (%)	659 (12.0)	538 (11.4)	121 (15.8)	.001
COPD, n (%)	1952 (35.6)	1695 (35.9)	257 (33.5)	.204
Liver disease, n (%)	525 (9.6)	419 (8.9)	106 (13.8)	<.001
Renal disease, n (%)	2544 (46.4)	2109 (44.7)	435 (56.7)	<.001
Malignant cancer, n (%)	520 (9.5)	388 (8.2)	132 (17.2)	<.001
Coronary artery disease, n (%)	2760 (50.3)	2345 (49.7)	415 (54.1)	.027
Cardiomyopathy, n (%)	1490 (27.2)	1274 (27.0)	216 (28.2)	.538
Implantable cardioverter-defibrillator pacemaker, n (%)	313 (5.7)	300 (6.4)	13 (1.7)	<.001
Percutaneous coronary intervention, n (%)	484 (8.8)	438 (9.3)	46 (6.0)	.004
Coronary bypass operation, n (%)	549 (10.0)	528 (11.2)	21 (2.7)	<.001
Used diuretic, n (%)	5041 (92.0)	4368 (92.6)	673 (87.7)	<.001
Used antiplatelet, n (%)	3925 (71.6)	3424 (72.6)	501 (65.3)	<.001
Used anticoagulant, n (%)	5190 (94.7)	4458 (94.5)	732 (95.4)	.353
Used statin, n (%)	3655 (66.7)	3177 (67.4)	478 (62.3)	.007
Used ACEI/ARB, n (%)	2448 (44.7)	2319 (49.2)	129 (16.8)	<.001
180-d readmission, n (%)	1398 (25.5)	1383 (29.3)	15 (2.0)	<.001
Death within 28 d, n (%)	767 (14.0)	0 (0.0)	767 (100.0)	<.001
Death within 90 d, n (%)	1250 (22.8)	483 (10.2)	767 (100.0)	<.001
Death within 180 d, n (%)	1531 (27.9)	764 (16.2)	767 (100.0)	<.001
Death within 365 d, n (%)	1913 (34.9)	1146 (24.3)	767 (100.0)	<.001

ACEI/ARB = angiotensin-converting enzyme inhibitor/angiotensin II receptor blocker, APS III = Acute Physiology Score III, COPD = chronic obstructive pulmonary disease, CRE = creatinine, ICU = intensive care unit, INR = international normalized ratio, OASIS = Oxford Acute Severity of Illness Score, PT = prothrombin time, PTT = partial thromboplastin time, RBC = red blood cell, RDW = red cell distribution width, SD = standard deviation, SIRS = systemic inflammatory response syndrome, SOFA = Sequential Organ Failure Assessment, WBC = White Blood Cell.

### 3.2. Association between SII and mortality in AHF patients

**Figure 2.**
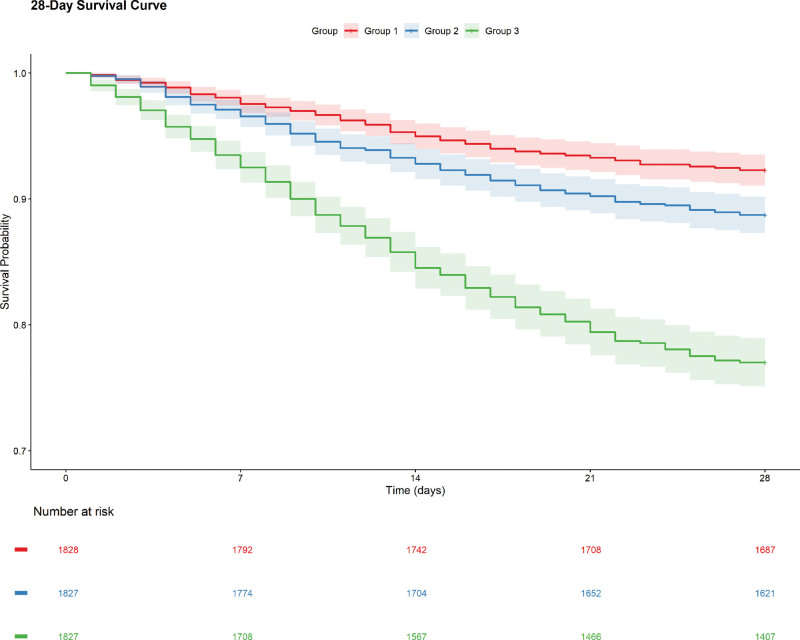
Kaplan–Meier (K–M) analysis of 28-day all-cause mortality stratified by systemic immune-inflammation index (SII) tertiles in acute heart failure (AHF) patients. Survival probability over 28 days is plotted, with the number at risk for each SII tertile group: group 1, group 2, and group 3.

Kaplan–Meier analysis revealed statistically significant disparities in 28-day all-cause mortality across SII tertiles (log-rank *P* < .001), with tertile 3 demonstrating markedly higher mortality versus lower tertiles (Fig. 2). Analogous statistical significance persisted for 90-, 180-, and 365-day mortality endpoints (Figures S1–S3, Supplemental Digital Content, https://links.lww.com/MD/R540).

**Figure 3.**
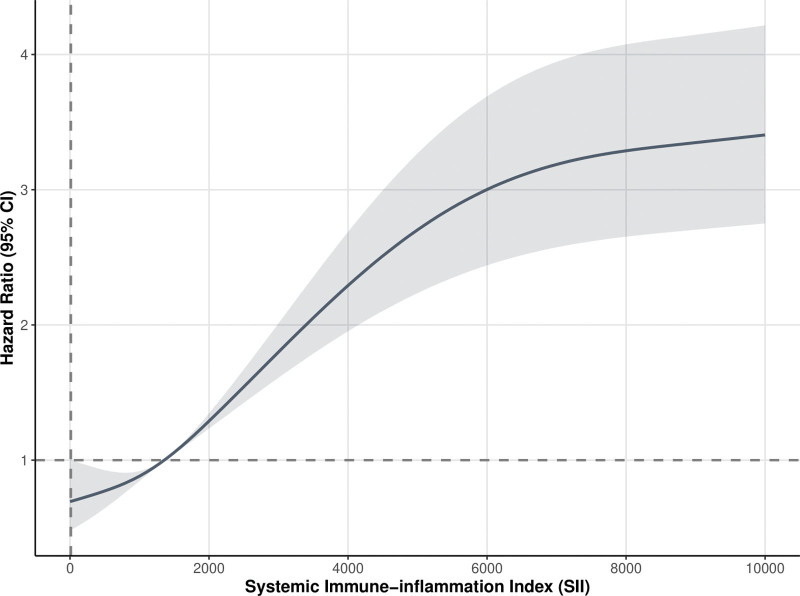
Dose–response relationship between SII and 28-day all-cause mortality in patients with AHF. RCS was adjusted for age, gender, coronary artery disease, diabetes mellitus, chronic obstructive pulmonary disease, myocardial infarction, atrial fibrillation, cardiomyopathy, chronic kidney disease, stroke, dyslipidemia, hypertension, liver disease, malignant cancers, creatinine, potassium, sodium, hemoglobin, platelets, leukocytes, erythrocyte distribution width, diuretics, ACEIs/ARBs, statins, antiplatelet agents, anticoagulants, coronary bypass graft/percutaneous coronary intervention, and implantable cardioverter-defibrillator pacemaker. ACEI/ARB = angiotensin-converting enzyme inhibitor/angiotensin II receptor blocker, AHF = acute heart failure, CI = confidence interval, RCS = restricted cubic spline, SII = systemic immune-inflammation index.

Multivariable Cox regression analyses (Table 3) identified graded mortality risks across SII tertiles, using tertile 1 as a reference. Tertile 2 exhibited significantly elevated 28-day mortality risk (hazard ratio [HR] = 1.485, 95% confidence interval [CI]: 1.193–1.849; *P* < .01), escalating further in tertile 3 (HR = 2.497, 95% CI: 2.060–3.028, *P* < .01). This dose–response pattern persisted across longer-term mortality endpoints (90-, 180-, and 365-day). In addition, a similar pattern was observed in the SII study of 90-, 180-, versus 365-day all-cause mortality, in which elevated levels of SII were associated with increased all-cause mortality (Table S1, Supplemental Digital Content, https://links.lww.com/MD/R540). RCS modeling confirmed continuous SII-mortality associations, demonstrating monotonically increasing 28-day mortality risks with rising SII levels after covariate adjustment (Fig. 3).

**Table 3:** Multivariable Cox regression analyses of the association between systemic immune-inflammation index (SII) tertiles and 28-day all-cause mortality in acute heart failure (AHF) patients.

	Model 1		Model 2		Model 3		Model 4	
	HR (95% CI)	*P*-value	HR (95% CI)	*P*-value	HR (95% CI)	*P*-value	HR (95% CI)	*P*-value
Q1	Ref		Ref		Ref		Ref	
Q2	1.489 (1.202, 1.844)	<.01	1.429 (1.153, 1.770)	<.01	1.409 (1.137, 1.746)	<.01	1.485 (1.193, 1.849)	<.01
Q3	3.245 (2.681, 3.927)	<.01	3.090 (2.553, 3.740)	<.01	2.979 (2.460, 3.609)	<.01	3.116 (2.532, 3.834)	<.01

Models include model 1 (unadjusted), model 2 (adjusted for age and sex), model 3 (additionally adjusted for comorbidities: coronary artery disease, diabetes mellitus, chronic obstructive pulmonary disease, myocardial infarction, atrial fibrillation, cardiomyopathy, chronic kidney disease, stroke, dyslipidemia, hypertension, liver disease, and malignant cancers), and model 4 (further adjusted for medications/interventions: diuretics, ACEI/ARB, statins, antiplatelet agents, anticoagulants, coronary artery bypass grafting/percutaneous coronary intervention, and implantable cardioverter-defibrillator/pacemaker). Hazard ratios (HR), 95% confidence intervals (CI), and *P*-values are reported, with SII tertile 1 as the reference, showing graded 28-day mortality risks across SII tertiles.

ACEI/ARB = angiotensin-converting enzyme inhibitor/angiotensin II receptor blocker, SII = systemic immune-inflammation index.

**Figure 4.**
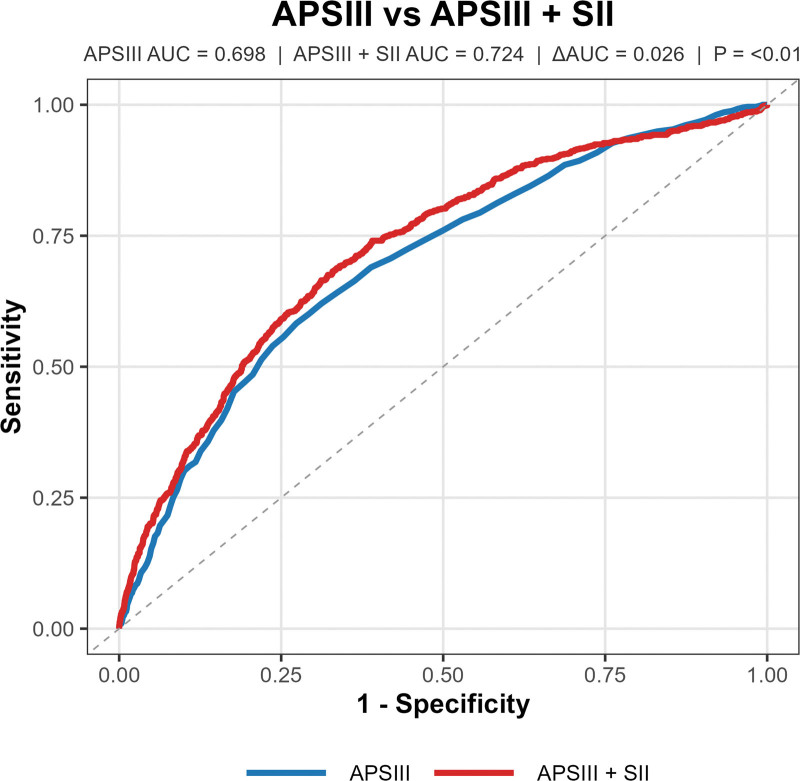
ROC curve analysis of APS III versus APS III + SII for predicting 28-day all-cause mortality in AHF patients, illustrating the predictive performance comparison between the Acute Physiology Score III (APS III) alone and APS III combined with SII. APS III has an AUC of 0.698, and APS III + SII achieves an AUC of 0.724, showing a significant incremental value (ΔAUC = 0.026, *P* < .01). AUC = area under the curve, ROC = receiver operating characteristic, SII = systemic immune-inflammation index.

**Figure 5.**
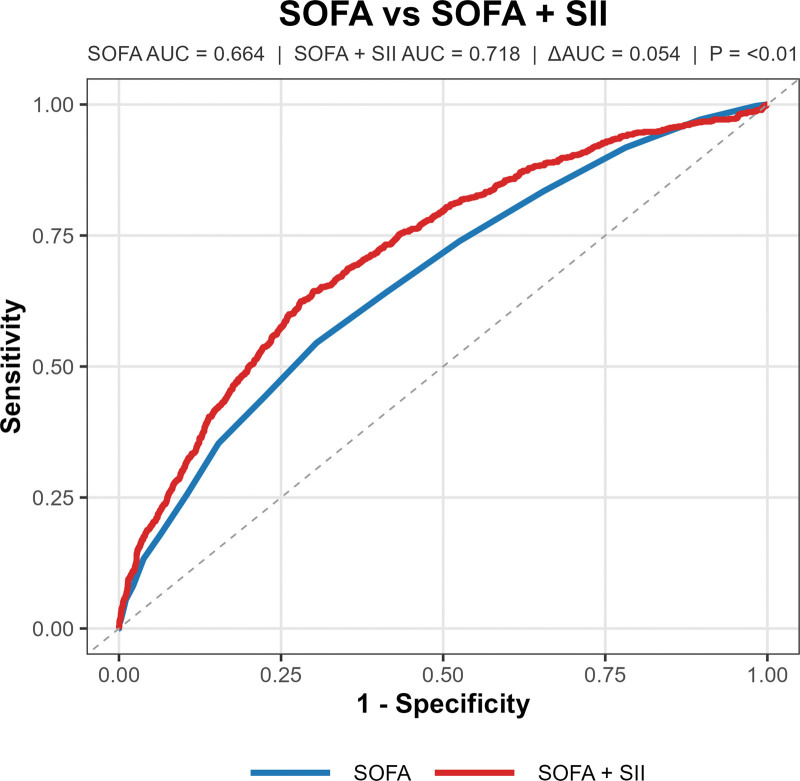
ROC curve analysis of SOFA versus SOFA + SII for predicting 28-day all-cause mortality in AHF patients, depicting the comparison of predictive performance between the Sequential Organ Failure Assessment (SOFA) score alone and SOFA combined with the systemic immune-inflammation index (SII). The area under the curve (AUC) for SOFA is 0.664, while SOFA + SII yields an AUC of 0.718, with a statistically significant increase (ΔAUC = 0.054, *P* < .01). AHF = acute heart failure, ROC = receiver operating characteristic.

To evaluate the incremental predictive value of SII beyond established risk stratification tools, we compared the performance of SOFA and APS III alone with their combinations with SII using receiver operating characteristic curve analysis for predicting 28-day all-cause mortality. For APS III, the area under the curve (AUC) was 0.698. When SII was added to APS III (APS III + SII), the AUC increased to 0.724, with a statistically significant ΔAUC of 0.026 (*P* < .01; Fig. 4). For SOFA, the AUC was 0.664 when used alone. Combining SOFA with SII (SOFA + SII) resulted in an AUC of 0.718, and the ΔAUC was 0.054 (*P* < .01; Fig. 5). These findings suggest that SII provides additional predictive information when integrated with SOFA or APS III for mortality risk stratification in AHF patients.

### 3.3. Subgroup analyses

**Figure 6.**
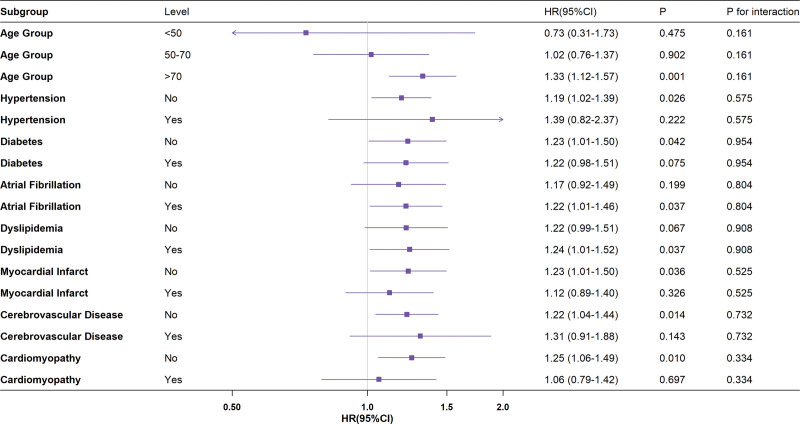
Association between different characteristics of SII and 28-day all-cause mortality in patients with AHF. AHF = acute heart failure, CI = confidence interval, HR = hazard ratio, SII = systemic immune-inflammation index.

Figure 6 presents stratified analyses evaluating the association between SII and 28-day mortality across clinical subgroups. Overall positive correlations persisted, and heterogeneity in HRs was observed across strata. In the age-stratified analysis, a significant elevation in mortality was noted in patients >70 years (HR = 1.33, 95% CI: 1.12–1.57; *P* = .001). By contrast, nonsignificant trends were observed in the <50-year (HR = 0.73, 95% CI: 0.31–1.73; *P* = .475) and 50- to 70-year (HR = 1.02, 95% CI: 0.76–1.37; *P* = .902) subgroups. For comorbidity interaction analyses, comparable effect magnitudes were identified between the hypertension group (HR = 1.39, 95% CI: 0.82–2.37; *P* = .222) and the nonhypertension group (HR = 1.19, 95% CI: 1.02–1.39; *P* = .036), as well as between the diabetes group (HR = 1.22, 95% CI: 0.89–1.51; *P* = .075) and the nondiabetes group (HR = 1.23, 95% CI: 1.01–1.50; *P* = .042). The nonsignificant interaction *P*-values (.575 and .954, respectively) indicate no evidence of effect modification in these cases. Notably, patients with atrial fibrillation exhibited slightly stronger SII-mortality associations (HR = 1.22, 95% CI: 1.01–1.46; *P* = .037) compared with non-AF counterparts (HR = 1.17, 95% CI: 0.92–1.49; *P* = .199). While a history of myocardial infarction appeared to slightly attenuate SII-mortality associations (HR = 1.12, 95% CI: 0.89–1.40; *P* = .326) compared with the non-myocardial infarction group (HR = 1.23, 95% CI: 1.01–1.50; *P* = .036), the interaction *P*-value (0.525) indicated no statistically significant modification. Similarly, subgroups with cerebrovascular disease (*P* = .732) and cardiomyopathy (*P* = .334) failed to show significant effect modifications. No statistically significant interactions were observed across the examined subgroups (all interaction *P*-values > .05).

### 3.4. Sensitivity analyses

To further validate the robustness of our core findings, we conducted a dedicated sensitivity analysis aimed at addressing potential confounding by infectious comorbidities and immunomodulatory treatments – factors that might independently influence both SII levels and mortality risk in AHF. Specifically, this analysis excluded 2 key subgroups: patients with a diagnosis of infectious conditions (including pneumonia and sepsis) and those receiving immunomodulatory medications (such as glucocorticoids, calcineurin inhibitors, and biologic agents targeting immune pathways). After these exclusions, the association between SII and 28-day all-cause mortality in the remaining AHF cohort remained statistically significant and consistent in direction with our primary analysis. Notably, higher SII levels continued to be strongly linked to an increased risk of death, with HRs maintaining a similar magnitude to those observed in the full cohort. The association between SII and 28-day all-cause mortality remained statistically significant after these exclusions (Table S2, Supplemental Digital Content, https://links.lww.com/MD/R540).

## 4. Discussion

Our findings are consistent with recent studies demonstrating the prognostic significance of SII in cardiovascular diseases. Prior investigations have shown that elevated SII predicts ventricular remodeling after myocardial infarction, new-onset HF following ST-segment elevation myocardial infarction, and adverse cardiovascular outcomes in coronary artery disease and HF cohorts.^[10–12,14]^ Extending these observations, our study provides novel evidence supporting the predictive value of SII in short-term mortality among critically ill AHF patients.

Our analysis of 5482 cases from the MIMIC-IV database revealed that patients in the highest SII tertile (>2118.25) faced a 2.497-fold increased 28-day mortality risk (95% CI: 2.060–3.028) compared with the lowest tertile, with consistent associations observed across 90-, 180-, and 365-day follow-ups. The prognostic value of SII – a composite metric integrating neutrophils, lymphocytes, and platelets – likely stems from synergistic pathophysiological mechanisms. Neutrophils induce myocardial damage through myeloperoxidase release and neutrophil extracellular trap formation while amplifying pro-inflammatorycascades via interleukin-6 and tumor necrosis factor-alpha.^[16,17]^ Recent mechanistic and clinical studies suggest that SII reflects the interplay between neutrophil-driven inflammation, lymphocyte-mediated immune suppression, and platelet activation, which jointly contribute to coronary lesion severity and cardiovascular risk.^[18]^ Lymphopenia reflects compensatory immunosuppression, impairing myocardial repair capacity.^[19]^ Platelets exacerbate ventricular remodeling through CD40 ligand-mediated endothelial activation beyond thrombotic effects.^[20]^ Notably, our findings revealed significant positive correlations between SII and oxidative stress markers (RDW, anion gap; *P* < .001), suggesting that SII may potentiate myocardial injury via redox imbalance. These observations align with recent single-cell sequencing studies demonstrating extensive neutrophil infiltration and complex immune-inflammatory interplay in AHF myocardial tissue.^[21]^ The tertile division of SII was guided by RCS analysis, which confirmed a nonlinear dose–response relationship between continuous SII and mortality (*P* for nonlinearity < .001). The cutoffs (Q1 ≤ 886.57; Q2 ≤ 2118.25; Q3 > 2118.25) aligned with natural inflection points in the RCS curve, where mortality risk accelerated significantly. This ensures the grouping captures clinically meaningful risk stratification rather than representing an arbitrary division.

Compared with conventional inflammatory markers, SII offers 3 distinct advantages. First, its cost-effectiveness and rapid accessibility make it highly suitable for emergency triage. SII is derived from conventional complete blood cell count parameters (neutrophils, platelets, and lymphocytes), making it a cost-effective and quickly available emergency triage tool. Biomarkers such as N-terminal pro-B-type natriuretic peptide (NT-proBNP) or high-sensitivity C-reactive protein (CRP) require specialized detection methods and are more costly. Second, SII holistically captures the inflammation-coagulation-immune axis through its tripartite cellular integration, surpassing the limited scope of singular markers such as CRP or the neutrophil-to-lymphocyte ratio.^[8]^ For instance, Kokubu et al demonstrated that high SII at discharge (>536 × 10^9^/L) was significantly associated with a composite endpoint of all-cause death and rehospitalization for worsening HF in AHF patients with preserved ejection fraction (HR = 1.28, 95% CI: 1.04–1.57, *P* = .020), even after adjusting for NT-proBNP and CRP, highlighting its independent prognostic value.^[22]^ By contrast, CRP’s prognostic significance often diminishes after multivariate adjustments. Third, SII’s prognostic superiority is supported by its sensitivity to systemic stress, which may not be fully captured by conventional markers such as NT-proBNP, which primarily reflect myocardial strain. Nguyen et al found that an NT-proBNP cutoff of ≥1858 pg/mL predicted 90-day events in HF with reduced ejection fraction (HR = 2.36, 95% CI: 1.31–4.24), but its specificity is limited in patients with renal dysfunction or atrial fibrillation, common AHF comorbidities.^[23]^ SII, derived from hematological parameters, maintains applicability across diverse clinical scenarios and reflects systemic inflammatory and thrombotic dynamics. For example, a study by Leal-Alcántara et al showed that the NT-proBNP/chloride ratio, which incorporates NT-proBNP, outperformed NT-proBNP alone in predicting cardiovascular events in AHF (HR = 3.18, 95% CI: 1.55–6.52, *P* = .0015), suggesting that combining biomarkers with systemic indicators enhances prognostic accuracy.^[24]^ SII’s inclusion of platelet counts adds a thrombotic dimension absent in the neutrophil-to-lymphocyte ratio, which only accounts for neutrophil and lymphocyte ratios, making SII a more comprehensive marker in AHF where thrombosis and inflammation interplay.^[25]^

Clinically, SII may serve as a readily available biomarker for early risk stratification in patients with AHF, particularly in emergency and critical care settings. Integrating SII with established clinical scoring systems may help improve short-term prognostic assessment.^[26]^ Several limitations of this study should be acknowledged. First, this was a single-center retrospective analysis, and residual confounding due to unmeasured variables cannot be fully excluded, including the lack of systematically available natriuretic peptides such as NT-proBNP. Second, SII was assessed only at the time of ICU admission, and longitudinal inflammatory trajectories were not evaluated. Third, other emerging inflammatory or fibrosis-related biomarkers (e.g., galectin-3 and soluble ST2) were not available for comparison in the present database.^[27,28]^ Finally, given the observational nature of the study, the findings reflect association rather than causation. Future studies with prospective and multicenter designs are warranted to validate these findings. Further investigations incorporating detailed HF phenotyping and serial inflammatory measurements may help clarify the clinical utility and biological relevance of SII in AHF.

Beyond its prognostic significance, the SII may offer insights into AHF’s broader systemic ramifications beyond cardiovascular pathology. AHF manifests as a syndromic condition precipitating multiorgan physiological derangements, including renal dysfunction, hepatic congestion, and metabolic dysregulation.^[29,30]^ Elevated SII levels may serve as sentinel markers of these interorgan crosstalk, reflecting the magnitude of systemic stress and inflammatory burden induced by AHF. For instance, neutrophil activation and platelet dysfunction – both captured by SII – are established mediators of microvascular injury and impaired organ perfusion, frequently observed in severe AHF presentations.^[31,32]^ Investigation of these interorgan relationships could deepen understanding of how SII integrates cardiac and extracardiac pathophysiology, potentially guiding more holistic management approaches.

Another critical consideration lies in SII’s potential for longitudinal therapeutic monitoring in AHF. While our study focused on baseline SII values at admission, serial SII measurements during hospitalization could elucidate trends associated with treatment response or disease progression. For example, SII reductions following diuretic therapy or anti-inflammatory interventions may indicate resolution of acute inflammatory states, whereas persistently elevated levels might signal refractory disease or complications such as superimposed infections.^[33–35]^ These temporal dynamics warrant dedicated investigation, as they could transform SII from a static prognostic marker to a real-time indicator for therapeutic strategy adjustments – a particularly valuable application in critical care settings requiring rapid clinical decision-making.

Finally, the integration of SII into established AHF risk prediction models merits exploration. Current scoring systems, such as the Guidelines for Heart Failure Management Risk Score or Acute Decompensated Heart Failure National Registry model,^[36,37]^ predominantly rely on clinical variables including blood pressure, renal function, and natriuretic peptide levels. Incorporating SII could enhance predictive accuracy by introducing currently overlooked inflammatory and immunological dimensions, particularly beneficial for stratifying intermediate-risk patients where traditional models lack precision. Collaborative efforts across multicenter cohorts could standardize SII integration into such frameworks, paving the way for its broader adoption in clinical guidelines and practice.

## 5. Conclusions

This study establishes SII as an independent predictor of 28-day all-cause mortality in AHF patients, demonstrating a robust dose–response relationship. Elevated SII (>2118.25) triples short-term mortality risk, unaffected by baseline comorbidities. By encapsulating innate immune activation, adaptive immunosuppression, and thromboinflammatory crosstalk, SII provides multidimensional prognostic insights surpassing conventional biomarkers, informing risk stratification, and paving the way for targeted immunomodulatory therapies. We advocate incorporating SII into emergency AHF assessment protocols and initiating interventional trials evaluating SII-guided personalized treatment paradigms.

## Author contributions

**Conceptualization:** Chengyu Zhang, Jiangan Li.

**Methodology:** Ziqi Zhang, Zi Yan, HangKai Cui.

**Supervision:** Ziqi Zhang, Xiaonan Ji.

**Investigation:** Zi Yan.

**Validation:** Xiaonan Ji.

**Resources:** Jiangan Li.

**Data curation:** Chengyu Zhang.

**Formal analysis:** Chengyu Zhang, Zi Yan.

**Project administration:** Chengyu Zhang, Jiangan Li.

**Software:** Chengyu Zhang, Ziqi Zhang, HangKai Cui, Jiangan Li.

**Writing – original draft:** Chengyu Zhang.

**Writing – review & editing:** Chengyu Zhang, Zi Yan, Jiangan Li.

## Supplementary Material
