# Supplementary material for: Association of systemic immune-inflammation index (SII) with 28-day all-cause mortality in acute heart failure: A retrospective cohort study
Source: Medicine (Baltimore). 2026 Mar 13;105(11):e48027. doi: 10.1097/MD.0000000000048027 (PMC12991674; doi:10.1097/MD.0000000000048027)
Supplement: Supplementary file 1 [file medi-105-e48027-s001.docx]

|  | Model1 | | Model2 | | Model3 | | Model4 | |
| --- | --- | --- | --- | --- | --- | --- | --- | --- |
|  | HR（95%CI） | P-Value | HR（95%CI） | P-Value | HR（95%CI） | P-Value | HR（95%CI） | P-Value |
| group1 | REF | | REF | | REF |  | REF |  |
| group2 | 1.489(1.276,1.759) | <0.01 | 1.444(1.230,1.696) | <0.01 | 1.410(1.201,1.657) | <0.01 | 1.325(1.128,1.557) | <0.01 |
| group3 | 2.892(2.499,3.347) | <0.01 | 2.788(2.409,3.227) | <0.01 | 2.681(2.315,3.104) | <0.01 | 2.305(1.989,2.672) | <0.01 |

Supplement Table 1

Relationship between SII and 90-day all-cause mortality in patients with AHF.

Relationship between SII and 180-day all-cause mortality in patients with AHF.

|  | Model1 | | Model2 | | Model3 | | Model4 | |
| --- | --- | --- | --- | --- | --- | --- | --- | --- |
|  | HR（95%CI） | P-Value | HR（95%CI） | P-Value | HR（95%CI） | P-Value | HR（95%CI） | P-Value |
| group1 | REF | | REF | | REF |  | REF |  |
| group2 | 1.446(1.257,1.663) | <0.01 | 1.400(1.217,1.661) | <0.01 | 1.363(1.185,1.568) | <0.01 | 1.269(1.102,1.460) | <0.01 |
| group3 | 2.537(2.230,2.887) | <0.01 | 2.461(2.163,2.801) | <0.01 | 2.367(2.079,2.695) | <0.01 | 2.033(1.784,2.317) | <0.01 |

Relationship between SII and 365-day all-cause mortality in patients with AHF.

|  | Model1 | | Model2 | | Model3 | | Model4 | |
| --- | --- | --- | --- | --- | --- | --- | --- | --- |
|  | HR（95%CI） | P-Value | HR（95%CI） | P-Value | HR（95%CI） | P-Value | HR（95%CI） | P-Value |
| group1 | REF | | REF | | REF |  | REF |  |
| group2 | 1.404(1.243,1.585) | <0.01 | 1.365(1.209,1.542) | <0.01 | 1.326(1.174,1.498) | <0.01 | 1.241(1.098,1.403) | <0.01 |
| group3 | 2.293(2.047,2.570) | <0.01 | 2.237(1.997,2.507) | <0.01 | 2.164(1.930,2.426) | <0.01 | 1.881(1.676,2.111) | <0.01 |

Supplementary Table 1 Multivariable Cox regression analyses of the association between Systemic Immunoinflammatory Index (SII) tertiles and 90-day, 180-day, 365-day all-cause mortality in acute heart failure (AHF) patients. Models: Model 1 (unadjusted); Model 2 (age/sex-adjusted); Model 3 (additional comorbidity adjustment); Model 4 (medication/intervention adjustment). Hazard ratios (HR), 95% confidence intervals (CI), and P-values are reported, with SII Tertile 1 (Group 1) as reference. Graded mortality risks across SII tertiles are observed for all endpoints (all P<0.01). SII=Systemic Immunoinflammatory Index; AHF=Acute Heart Failure; HR=Hazard Ratio; CI=Confidence Interval.

Supplement Table 2:

Sensitivity Analysis: Assessing the Independence of SII's Prognostic Value in Acute Heart Failure (AHF) from Infectious and Immunomodulatory Confounders.

|  | Model1 | | Model2 | | Model3 | | Model4 | |
| --- | --- | --- | --- | --- | --- | --- | --- | --- |
|  | HR（95%CI） | P-Value | HR（95%CI） | P-Value | HR（95%CI） | P-Value | HR（95%CI） | P-Value |
| group1 | 0.318(0.235, 0.431) | <0.01 | 0.322(0.238, 0.436) | <0.01 | 0.318(0.234, 0.431) | <0.01 | 0.359(0.264, 0.489) | <0.01 |
| group2 | 0.397(0.300, 0.526) | <0.01 | 0.406(0.306, 0.538) | <0.01 | 0.412(0.311, 0.552) | <0.01 | 0.451(0.340, 0.601) | <0.01 |
| group3 |  | REF |  | REF |  | REF |  | REF |

Supplementary Table 2 presents the results of a sensitivity analysis conducted to determine the robustness of the association between Systemic Immunoinflammatory Index (SII) and 28 - day all - cause mortality in acute heart failure (AHF) patients. This analysis excluded patients with infectious comorbidities (such as pneumonia and sepsis) and those on immunomodulatory medications (including glucocorticoids, calcineurin inhibitors, and biologic agents targeting immune pathways). Hazard ratios (HRs) and 95% confidence intervals (CIs) from multivariable Cox regression models are reported. The data demonstrate that, even after excluding these potentially confounding subgroups, higher SII levels remain significantly associated with an increased risk of 28 - day all - cause mortality, indicating the independent prognostic value of SII in AHF. SII = Systemic Immunoinflammatory Index; AHF = Acute Heart Failure; HR = Hazard Ratio; CI = Confidence Interval.

Supplementary Figures

Supplementary Figure 1 Kaplan - Meier (KM) analysis of 90 - day all - cause mortality stratified by SII tertiles in AHF patients. The plot depicts survival probability across 90 days, accompanied by the number at risk for each of the three SII tertile groups (Group 1, Group 2, Group 3) at specified time points.





Supplementary Figure 2 Kaplan - Meier (KM) analysis of 180 - day all - cause mortality stratified by SII tertiles in AHF patients. Survival probability trends over 180 days are illustrated, with the number at risk for Group 1, Group 2, and Group 3 provided to reflect sample size dynamics during the follow - up.





Supplementary Figure 3 Kaplan - Meier (KM) analysis of 365 - day all - cause mortality stratified by SII tertiles in AHF patients. This figure presents the 1 - year survival probability patterns, along with the number at risk for each SII tertile group, enabling assessment of long - term mortality differences associated with SII levels.
